# Deciphering decidual leukocyte traffic with serial intravascular staining

**DOI:** 10.3389/fimmu.2023.1332943

**Published:** 2024-01-10

**Authors:** Jessica Vazquez, Mona A. Mohamed, Soma Banerjee, Logan T. Keding, Michelle R. Koenig, Fernanda Leyva Jaimes, Rachel C. Fisher, Emily M. Bove, Thaddeus G. Golos, Aleksandar K. Stanic

**Affiliations:** ^1^ Department of Obstetrics and Gynecology, University of Wisconsin-Madison, Madison, WI, United States; ^2^ Wisconsin National Primate Research Center, Madison, WI, United States; ^3^ Department of Comparative Biosciences, University of Wisconsin-Madison, Madison, WI, United States

**Keywords:** decidua, leukocytes, traffic, gestation, non-human primate, murine, T cells, ILCs

## Abstract

The decidual immunome is dynamic, dramatically changing its composition across gestation. Early pregnancy is dominated by decidual NK cells, with a shift towards T cells later in pregnancy. However, the degree, timing, and subset-specific nature of leukocyte traffic between the decidua and systemic circulation during gestation remains poorly understood. Herein, we employed intravascular staining in pregnant C57BL/6J mice and cynomolgus macaques (*Macaca fascicularis*) to examine leukocyte traffic into the decidual basalis during pregnancy. Timed-mated or virgin mice were tail-vein injected with labelled αCD45 antibodies 24 hours and 5 minutes before sacrifice. Pregnant cynomolgus macaques (GD155) were infused with labelled αCD45 at 2 hours or 5 mins before necropsy. Decidual cells were isolated and resulting suspensions analyzed by flow cytometry. We found that the proportion of intravascular (IVAs)-negative leukocytes (cells labeled by the 24h infusion of αCD45 or unlabeled) decreased across murine gestation while recent immigrants (24h label only) increased in mid- to late-gestation. In the cynomolgus model our data confirmed differential labeling of decidual leukocytes by the infused antibody, with the 5 min infused animal having a higher proportion of IVAs+ cells compared to the 2hr infused animal. Decidual tissue sections from both macaques showed the presence of intravascularly labeled cells, either in proximity to blood vessels (5min infused animal) or deeper into decidual stroma (2hr infused animal). These results demonstrate the value of serial intravascular staining as a sensitive tool for defining decidual leukocyte traffic during pregnancy.

## 1 Introduction

The maternal immune system plays a critical role at the maternal-fetal interface by providing protection against pathogens (1), maintaining tolerance towards the semi-allogeneic fetus (2, 3), and promoting vascular remodeling in the decidua (4–6). The decidua (endometrium of pregnancy underlying the placenta and fetal membranes) is a specialized mucosa that maintains a dynamic immunome. In recent years, extensive research has been directed at developing a better understanding of the leukocyte composition at the decidua in humans, mice and rhesus macaque systems using advanced flow cytometry, mass cytometry and single cell sequencing (7–13). By virtue of experimental design and necessity, these studies have all relied on single time-point snapshots of immune composition at the maternal fetal interface (decidua and placenta), limiting our understanding of immune trafficking between systemic circulation and target tissue.

The composition of the decidual (human and mouse) immunome is specific to studied gestational time-points, as early pregnancy is dominated by decidual NK (dNK) cells, with few T cells observed (14–17). This pattern shifts in late pregnancy to one that is dominated by T cells (18). Early and mid-pregnancy is of particular interest as deficits in normal dNK cell-driven vascular remodeling result in a higher risk of preeclampsia and other pregnancy pathology (19–21). Some studies have suggested that conventional NK cells can acquire a “decidua-like” phenotype upon exposure to specific cytokine cocktails (22), while others suggest that CD34+ hematopoietic precursors differentiate in human decidua *in situ* (23). In recent studies of dNK cells, at least one subset appears to be tissue resident (24) in a murine parabiosis model, while others might traffic into the decidua and/or expand locally in pregnancy. The question of decidual lymphocyte traffic is especially relevant as human studies suggest that as many as three dNK cells subsets exist, distinguished by cell surface receptors and transcriptional/functional programming (13). Similarly, innate lymphoid cells (ILCs) have been identified in both the murine (11, 25, 26) and human decidua (8, 27, 28) and have been associated with pre-term birth (29, 30). Although ILCs are found in circulation, they are most abundant at mucosal sites (31, 32). And, like NK cells, many novel subsets have been identified in the decidua (33, 34), however, questions remain as to whether they are recruited from the circulation or whether they proliferate and differentiate *in situ.*

T cells and antigen presenting cells (APCs) play a critical role in protection of and tolerance to the conceptus at the maternal-fetal interface (1, 18). From the first trimester onward, there is a gradual and progressive change in the frequency and functionality of both T and immune myeloid cells (monocytes, macrophages, dendritic cells, and granulocytes) as pregnancy progresses. Whereas T cell frequency, especially effector T cells, increases dramatically toward term, the frequency of myeloid cells remains relatively high and constant across gestation (9, 15, 35, 36). However, the origin of these gestational shifts in immune cell frequency, whether due to trafficking, differentiation or cell proliferation is unclear. It has been suggested that epigenetic regulation of chemokine expression is the primary mechanism by which entrance of T cells and exit of dendritic cells is regulated (18, 37, 38). Similarly, chemokines have also been implicated in recruitment of mucosal-associated invariant T (MAIT) cells into the intervillous space (39).

Taken together, a comprehensive model of how decidual leukocytes traffic, expand and contract in numbers during pregnancy and the peripartum period is still elusive. Development and testing of approaches to simultaneously track cellular trafficking, differentiation and function of decidual immune cells is essential for deciphering the role of the immune system in pregnancy pathology. Multiple techniques have been developed to understand how leukocytes traffic across various tissues (40, 41), such as the use of parabionts (24, 41), adoptive transfer (42) and *in vivo* homing assays (43), and intravital microscopy (40, 44). CFSE injection directly into the cervix has also been used to assess migration of dendritic cells in the decidua (36), although this technique only allows for a one timepoint measurement. As a means to start delineating traffic of individual cell types in the decidua, we employed serial intravascular staining (SIVS), a powerful method technique by which to assess tissue localization and traffic of lymphocytes (45–48). We illustrate that a similar approach is possible in both the mouse and non-human primates to understand leukocyte traffic in the decidua during pregnancy. Our data indicate that this technique will prove invaluable in our understanding of leukocyte traffic in the decidua, with wide applications, including integration with infectious model studies.

## 2 Materials and methods

### 2.1 Ethics, care & use of macaques

The rhesus macaques used in this study were cared for by the staff at the Wisconsin National Primate Research Center (WNPRC) according to regulations and guidelines of the University of Wisconsin Institutional Animal Care and Use Committee, which approved this study protocol (G006070) in accordance with recommendations of the Weatherall report and according to the principles described in the National Research Council’s Guide for the Care and Use of Laboratory Animals. All animals were housed in enclosures with at least 4.3, 6.0, or 8.0 sq. ft. of floor space, measuring 30, 32, or 36 inches high, and containing a tubular PVC or stainless steel perch. Each individual enclosure was equipped with a horizontal or vertical sliding door, an automatic water lixit, and a stainless steel feed hopper. All animals were fed using a nutritional plan based on recommendations published by the National Research Council. Twice daily, macaques were fed a fixed formula of extruded dry diet (2050 Teklad Global 20% Protein Primate Diet) with adequate carbohydrate, energy, fat, fiber (10%), mineral, protein, and vitamin content. Dry diets were supplemented with fruits, vegetables, and other edible foods (e.g., nuts, cereals, seed mixtures, yogurt, peanut butter, popcorn, marshmallows, etc.) to provide variety to the diet and to inspire species-specific behaviors such as foraging. To further promote psychological well-being, animals were provided with food enrichment, human-to-monkey interaction, structural enrichment, and manipulanda. Environmental enrichment objects were selected to minimize chances of pathogen transmission from one animal to another and from animals to care staff. While in the study, all animals were evaluated by trained animal care staff at least twice daily for signs of pain, distress, and illness by observing appetite, stool quality, activity level, and physical condition. Animals exhibiting abnormal presentation for any of these clinical parameters were provided appropriate care by attending veterinarians.

The female macaques described in this report were co-housed with a compatible male and observed daily for menses and breeding. Pregnancy was detected by abdominal ultrasound, and gestational age was estimated as previously described (49). For physical examinations, antibody infusions, and blood collections, dams were anesthetized with an intramuscular dose of ketamine (10 mg/kg). Blood samples from the femoral or saphenous vein were obtained using a vacutainer system or needle and syringe. Pregnant macaques were monitored daily prior to and after infusions to assess general well-being and for any clinical signs of distress.

### 2.2 Intravascular staining

**Figure 1:**
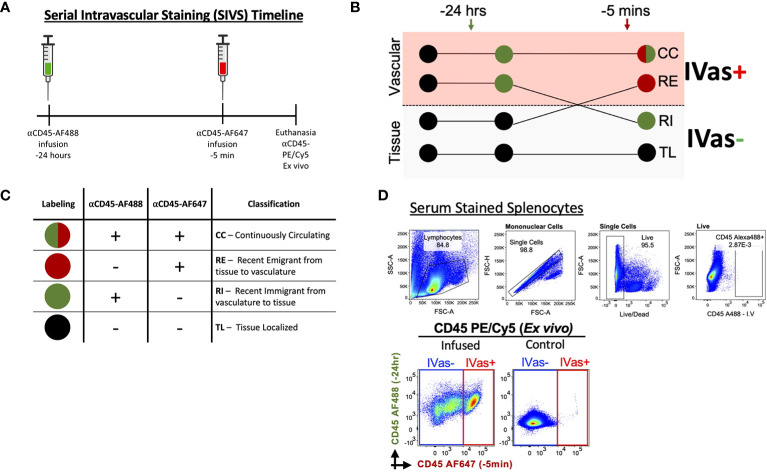
Serial Intravascular Staining of Leukocytes in the Mouse. **(A)** Experimental timeline illustrating serial infusions of αCD45 fluorochrome conjugated antibodies. **(B)** Schematic illustrating labeling of leukocytes by infused antibodies and **(C)** Interpretation of resulting intravascular labeling. **(D)** *Top* Labeling of splenocytes with serum of animal infused with αCD45-AF488 24 hours before euthanasia (plots representative of 2 experiments); *Bottom* Plots comparing infused animal (left) and not infused animal (right). Plots representative of 5 experiments.

Female and male C57BL/6J (B6) mice were purchased from Jackson laboratory (Bar Harbor, ME). B6 female mice (6-13 weeks) were used for timed mating and experiments. The day when a vaginal plug was detected in a timed mating was counted as gestational day 0.5. Mice were tail-vein injected with 6.7 μg of AF488-conjugated CD45 antibody 24 hours and 6.7 μg of AF647-conjugated CD45 antibody 5 minutes before sacrifice (**Figure 1A**; **Supplementary Table 1**). Virgin mice and pregnant mice were sacrificed, and gestational day of each decidua was recorded. For technical calibration controls, spleens of uninfused wild-type mice were also collected.

Two pregnant (GD155) cynomolgus macaques (*Macaca fascicularis*) were infused with AF555-conjugated αCD45 (100 μg/kg) 2 hours and 5 mins before necropsy, respectively. Animals were then euthanized and decidua and placenta, as well as selected maternal tissues (spleen, lymph nodes), were collected. Peripheral blood was collected pre- and post-infusion.

### 2.3 Tissue processing

Mouse decidua or uteri were collected and minced with scissors in RMPI 1640 containing collagenase type V/DNAse I (Worthington Biochem). These specimens were then loaded in gentleMACS™ C tubes (Miltenyi Biotec Inc), and a specially adapted tissue dissociation program was run in a gentleMACS™ Dissociator for 30 minutes (Miltenyi Biotec Inc) (9). Spleen and Peyer’s patches (used as vascularly-permeant and mucosal lymphoid control tissues, respectively) were mechanically dissociated in RMPI 1640 containing 10% heat-inactivated FBS in gentleMACS™ C tubes by running corresponding programs for different tissue types in the gentleMACS™ Dissociator. After dissociation, homogenates were filtered through a 70 μm cell strainer, and red blood cells were lysed with ACK lysis buffer (Life Technologies). The resulting single cell suspensions were used in downstream flow cytometry applications.

Macaque decidua was dissected from the placenta and mononuclear cells were isolated as previously described (7). Maternal spleen and lymph nodes were processed as described above. Peripheral mononuclear cells (PBMCs) were isolated by gradient centrifugation. Briefly, blood was layered on Ficoll gradient media, spun, and the buffy coat was collected into RPMI. PBMCs were then washed and frozen for later analysis. In addition, tissue biopsies were collected and fixed overnight in 4% paraformaldehyde and paraffin embedded (FFPE). FFPE tissues sections (5 μm) were then stained with nuclear stain DAPI and imaged using the Nikon confocal microscope. Serial sections were stained with either hematoxylin and eosin and imaged using the Nikon Eclipse microscope.

### 2.4 Flow cytometry

Isolated MCs were first labeled with LIVE/DEAD**^®^** fixable blue stain (Invitrogen) according to manufacturer’s instructions. MCs were then labeled with fluorochrome-conjugated monoclonal antibodies, listed in **Supplementary Table 1**. Briefly, antibodies were diluted in BD Horizon Brilliant™ Stain Buffer (BD Biosciences, San Jose, CA) and used to label MCs according to the manufacturer’s instructions. Samples were then acquired using the LSR Fortessa in a 5 laser (355nm, 405nm, 488nm, 562nm, 633nm) 20-detector configuration (BD Biosciences) or in a Cytek Aurora spectral cytometer. Prior to downstream analysis, FCS files were either compensated within FlowJo or spectrally unmixed using the SpectroFlo software.

### 2.5 Data analysis

Manual analysis identifying well-characterized populations was performed using FlowJo v.10 software (FlowJo LLC, Ashland, OR). Dimensionality reduction was performed using the FlowSOM algorithm (50) and visualized within FlowJo. Briefly, Lineage negative, CD45-positive events were down sampled from 6 samples (2 virgin, 2 early-GD, 1 mid-GD, 1 late-GD), to 1000 cells per sample, for a total of 6000 cells (2 samples did not meet this threshold and were not down sampled). Down sampled populations were then coded for sample type and concatenated. The concatenated file was then analyzed using FlowSOM plugin within FlowJo, using only phenotypic marker expression (CD4, CD49a/b, CD103, KLRG1, and NK1.1). The resulting median fluorescence intensity (MFI) values for these markers were calculated within FlowJo for identified clusters. Cluster frequency was similarly calculated within FlowJo. Resulting data tables were coded and heatmap construction was done using the R package ComplexHeatmap (51). Clusters were annotated based on expression heatmaps, based on known expression markers (52, 53). All data are represented as median with interquartile range and statistical significance was determined by ANOVA, followed by Tukey’s *post-hoc* test to correct for multiple comparisons, using Prism**^®^** v. 7 (GraphPad Software, Inc, La Jolla, CA) or JMP® Pro v. 15.0.0 (SAS Institute Inc, Cary, NC), unless stated otherwise.

## 3 Results

### 3.1 Serial intravascular staining reveals leukocyte trafficking in mouse mucosal tissues

To determine if SIVS is a viable approach to studies of leukocyte traffic in virgin and pregnant mice we tested our ability to distinguish leukocytes isolated from uterus and decidua (**Figure 1A**). We maintained previously established nomenclature (45) and interpretation to determine whether leukocytes were designated as continuously circulating (CC), recent stromal emigrants (RE), recent immigrants (RI) to the virgin uterus/decidua, or tissue localized (TL) in the virgin uterus/decidua (**Figures 1B, C**). To exclude the possibility that unbound αCD45-AF488 would be circulating at the 5 min infusion time point, we tested the ability of serum from an infused animal to label splenocytes from a control animal and found that the serum did not label any splenocytes (**Figure 1D**, top), confirming that there was no unbound αCD45-AF488 capable of labeling leukocytes prior to euthanasia, with similar levels of background labeling compared to non-injected mice (**Figure 1D**, bottom).

### 3.2 Decidual trafficking of CD45+ leukocytes across pregnancy

**Figure 2:**
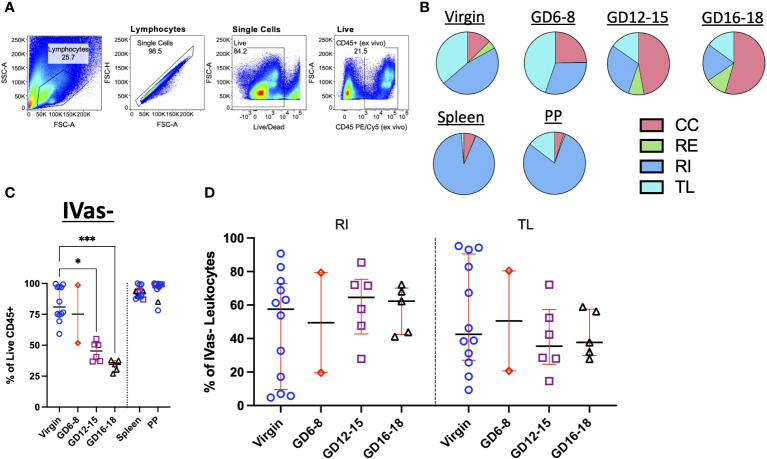
Trafficking Dynamics of Leukocytes Across Murine Gestation. **(A)** Representative gating strategy identifying total leukocytes in murine decidua. **(B)** Quantification of CC, RE, RI, TL leukocytes in virgin uterus, indicated gestational day (GD) decidua, spleen and Peyer’s patch (PP) of animals following infusion protocol outlined in **Figure 1A**. CC, continuously circulating; RE, recent emigrant; RI, recent immigrant; TL, tissue localized. Virgin, n = 5; GD6-8, n = 2; GD12-15, n = 3; GD16-18, n = 2. **(C)** Proportion of IVas- leukocytes from total leukocytes (Live CD45+) in the virgin uterus, early (GD6-8), mid (GD12-15), and late (GD16-18) murine gestation. Spleen and Peyer’s patch proportions also quantified for some animals. **(D)** Proportion of Recent Immigrant (RI) and Tissue Localized (TL) leukocytes from IVas- leukocytes across murine gestation. Shown are median with interquartile range. Virgin, n = 12; GD6-8, n = 2; GD12-15, n = 6; GD16-18, n = 5. Statistical significance was determined by One-way ANOVA followed by Tukey’s *post-hoc* test. *p < 0.05, ***p < 0.001.

We next assessed the trafficking status of total leukocytes in the decidua (**Figure 2A**). To determine if SIVS was capable of highlighting differences in leukocyte traffic across tissues, we assessed the proportion of CC, RE, RI, and TL leukocytes from our initial experiments and found that, indeed, we were able to detect increasing proportion of CC leukocytes in late gestation (**Figure 2B**, top). Furthermore, control tissues, Peyer’s patches (PPs) and spleen, chosen for relevance to pregnancy (virgin uterus, gestational decidua), a mucosal lymphocyte-rich tissue (PPs) and a vascular permeant tissue (spleen), displayed expected proportions of circulating leukocytes (47) (**Figure 2B**, bottom). Having established that the application of SIVS was capable of highlighting differences in the vascular status of leukocytes in murine pregnancy, we proceeded with a more detailed analysis of leukocyte traffic in the decidua (**Figures 2C, D**). First, we examined IVas- leukocytes, cells either labeled only by the 24h infusion of αCD45-AF488 or those that remained entirely unlabeled (**Figure 1B**) indicating inaccessibility to the intravascular compartment at the time of euthanasia. We found that the proportion of IVas- (RI + TL) leukocytes in the decidua decreased across gestation, while spleen and PP IVas- leukocyte proportions remained stable (**Figure 2C**). In agreement with other observations (54, 55), we found that there was an increase in the proportion of RI leukocytes in mid- to late-gestation (**Figure 2D**), suggesting an influx of leukocytes nearing parturition. A decrease in IVas- leukocytes conversely lead to a proportional increase in IVas+ (CC + RE) leukocytes (**Figure 2B**). This observation, coupled with a higher proportion of RI leukocytes suggests that there is increased leukocyte traffic in late gestation, supporting the concept that the decidua is actively restructured immunologically when nearing parturition (37, 54).

### 3.3 Gestational traffic of decidual T cells and innate lymphocytes and their subsets

**Figure 3:**
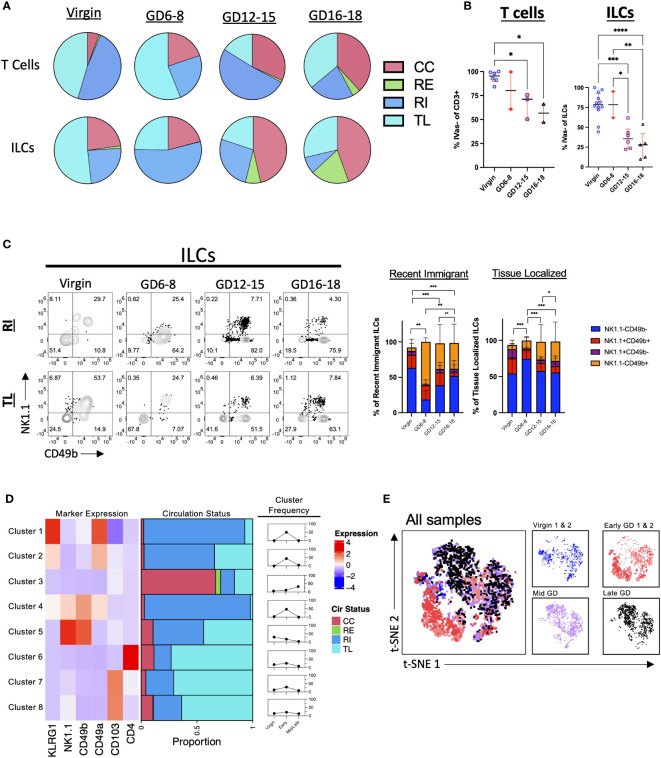
Pattern of T Cell and ILC Entrance and Exit Across Murine Gestation. **(A)** Quantification of CC, RE, RI, TL T cells and ILCs in virgin uterus and indicated GD decidua. Virgin, n = 5; GD6-8, n = 2; GD12-15, n = 3; GD16-18, n = 2. **(B)** Proportion of IVas- T cells (left) and ILCs (right). Shown are median with interquartile range. T cells: Virgin, n = 5; GD6-8, n = 2; GD12-15, n = 3; GD16-18, n = 2. ILCs: Virgin, n = 12; GD6-8, n = 2; GD12-15, n = 6; GD16-18, n = 5. Statistical significance was determined by One-way ANOVA followed by Tukey’s *post-hoc* test. *p < 0.05, **p < 0.005, ***p < 0.0005, ****p < 0.0001. **(C)** Distribution of NK1.1 and CD49b expression within RI and TL ILCs across gestation. Statistical significance was determined by Pearson’s chi-squared test followed by nonparametric Wilcoxon test. *p < 0.05, **p < 0.005, ***p < 0.0001. Virgin, n = 9; GD6-8, n = 2; GD12-15, n = 6; GD16-18, n = 5. **(D, E)** ILCs (pre-gated LiveCD45+CD3-TCRβ-CD11c-CD19-Ly6G-) from 2 virgin, 2 GD6-8 (Early), and 2 GD12-18 (Mid/Late) animals were clustered using FlowSOM **(D)** and visualized with t-SNE **(E)**. **(D)** *Left*, heatmap summarizing marker expression across the 8 clusters identified. *Center*, bar plot summarizing the proportion of CC, RE, RI, and TL ILCs within each cluster. *Right*, summarizing line graphs. **(E)** *Left*, t-SNE map overlayed with animals included in analysis. *Right*, t-SNE maps segregated by virgin/GD. CC, continuously circulating; RE, recent emigrant; RI, recent immigrant; TL, tissue localized.

Both adaptive and innate lymphocytes have been implicated in having important roles in pregnancy (1, 56, 57). To determine their respective trafficking dynamics, we assayed both the T cell compartment (CD3+) and Innate Lymphocytes (decidual NK cells and ILCs) in the murine decidua. Broadly, the results indicate an increase in CC and a decrease in TL cells in both T and ILC compartments across pregnancy (**Figure 3A**; **Supplementary Figure 1**). Closer examination of the data confirmed this trend, with the proportion of IVas- (RI + TL) cells decreasing in both T cells and ILCs (**Figure 3B**). Interestingly, the proportion of TL T cells increased in late gestation, suggesting that T cells enter and persist (>24h) in the decidua in late gestation as has been previously observed (18, 58), while the proportion of TL ILCs remained lower across gestation (**Figure 3A**; **Supplementary Figure 2**). The majority of gestational RE and TL ILCs were NK1.1- and CD49b-negative (**Figure 3C**), suggesting that they are not classical NK cells or trNK cells (24, 59). Furthermore, we saw an increase in the proportion of RI ILCs that were NK1.1-CD49b+ (**Figure 3C**) in early gestation, suggesting that this subset of ILCs is recruited into the decidua early in pregnancy.

To better understand decidual ILC trafficking dynamics, we employed FlowSOM (50), a dimensionality reduction technique that allows for the unsupervised clustering of flow cytometry data sets (**Supplementary Figure 3**), coupled with t-SNE visualization (60) A total of 6 samples (2 virgin, 2 GD6-8, 1 GD12-15, and 1 GD16-18) were used for clustering analysis. We instructed FlowSOM to partition our dataset into 8 meta clusters (**Figure 3D**; **Supplementary Figure 3** and **Supplementary Table 2**) based on marker expression. Only one cluster (Cluster 3; ILCs) had a majority of cells that were CC, with this cluster having a higher frequency in mid/late gestation (**Figures 3D, E**). Early pregnancy was dominated by Clusters 1, 2 (ILC2-like) and 4 (intermediate ILC1s), all CD49a+ and RI, suggesting that these subsets are recruited upon the initiation of decidualization in the mouse leading to upregulation of CD49 expression (**Figure 3D**). Indeed, we found that the early GD samples occupied an entire portion of the tSNE map, corresponding to cluster 4 (**Figure 3E**; **Supplementary Figure 3B**). These subsets can also represent cells that are closely associated with decidual vasculature and therefore are labeled by infused antibodies. Interestingly, TL ILCs were mostly non-NK ILCs expressing the tissue-residency marker CD103. Rather than a new decidual ILC (dILCs) subset, these TL ILCs might be in a state of transition, under the influence of the decidual stroma.

### 3.4 SIVS proof-of-concept in cynomolgus macaque decidua detected by flow cytometry

**Figure 4:**
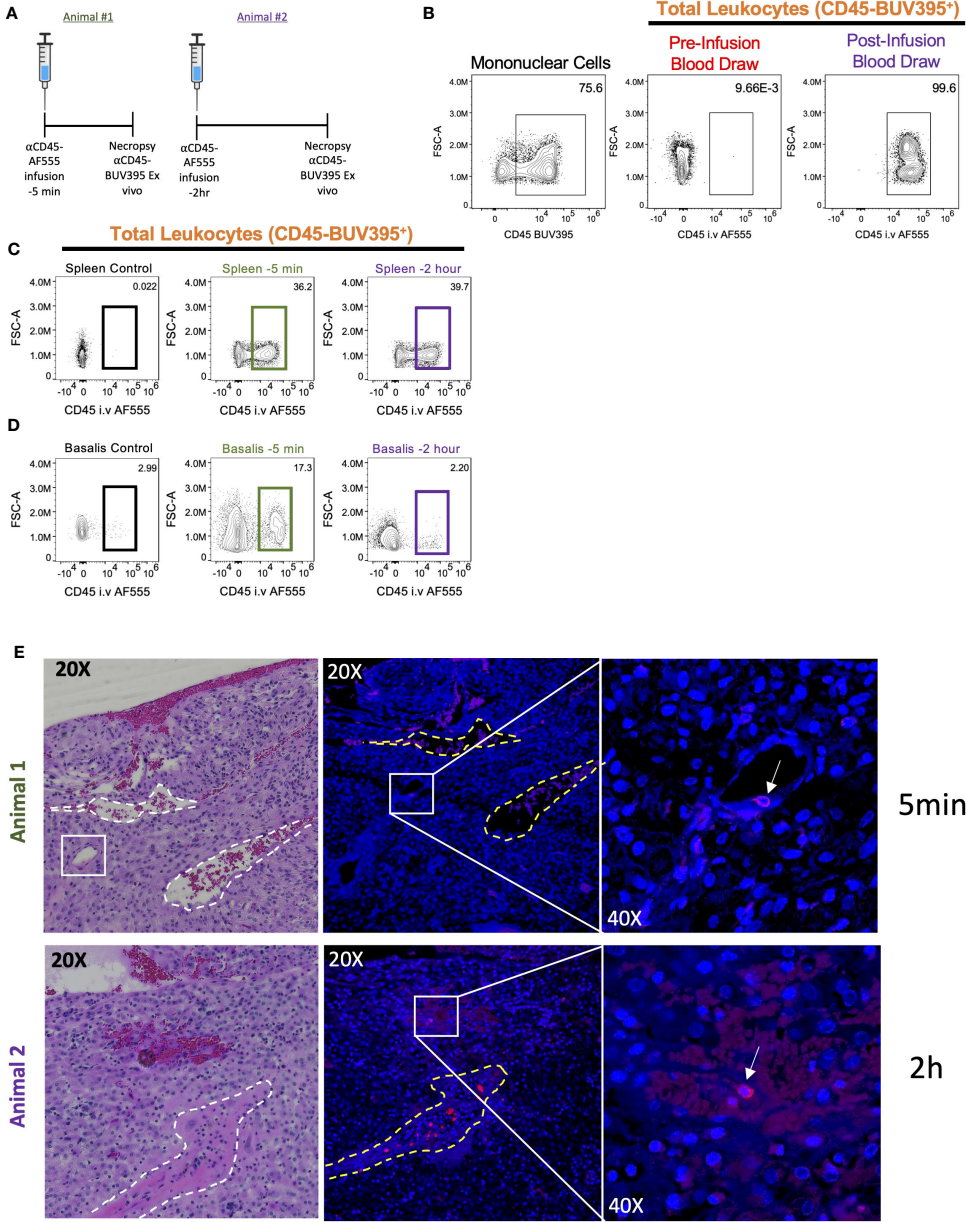
Intravascular staining of pregnant cynomolgus macaques. Two pregnant cynomolgus macaques were intravascularly labeled with αCD45 AF555 conjugated antibody 2 hours and 5 mins before necropsy, respectively. **(A)** Experimental timeline for each animal. **(B)** Representative gating scheme identifying total leukocytes in PBMCs before and after infusion. **(C, D)** Representative gating scheme identifying total leukocytes in the spleen **(C)** and **(D)** decidua. **(E)** Representative images of decidual sections. Serial sections from Animal 1 (*top*) – tissues collected 5 mins post-infusion – and Animal 2 (bottom) – tissues collected 2 hours post-infusion. H&E stained (left) section with two veins outlined (white dotted lines) and a blood vessel resembling a high endothelial venule (HEV) in white box. Fluorescent section stained with DAPI (middle) with vein outlines. Higher magnification (left) of middle panel showing a labeled leukocyte (white arrow).

The non-human primate (NHP) is the ideal model for human pregnancy, sharing many important characteristics, including placenta villous morphology, placental MHC and endocrine phenotypes, and degree of spiral artery remodeling (61) that is not seen in mice. Recently, serial intravascular staining has been shown to delineate trafficking of leukocytes in the rhesus macaque (45, 46). To provide preliminary evidence that the cynomolgus macaque decidua can be effectively analyzed using SIVS we designed a pilot experiment involving one antibody (αCD45-AF555) infusion into pregnant (GD155, term GD165) cynomolgus macaques at either 2 hours (Animal #2) or 5 mins (Animal #1) before necropsy (**Figure 4A**) to assess intravascular (i.v.) staining of leukocytes in the decidua. We confirmed successful antibody infusions by assessing PBMC αCD45-AF555 staining pre- and post-infusion (**Figure 4B**) and found that the majority of circulating PBMCs were indeed stained. Next, we assessed the proportion of leukocytes that were labeled in the spleen and decidua of both infused animals (**Figures 4C, D**). As expected, we found that a similar proportion of splenic leukocytes were labeled in both the 2hr and 5min animals (**Figure 4C**), with little αCD45-AF555 signal in a control (not infused) spleen. The decidua, however, displayed differential labeling, with the decidua from the 5min infused animal having a higher proportion of labeled cells compared to the 2hr infused animal (**Figure 4D**).

### 3.5 Detection of architectural localization of labeled lymphocytes in cynomolgus macaque decidual sections

To explore our ability to detect architectural localization of stained lymphocytes within decidual tissue, we examined FFPE sections of this tissue from both animals. These demonstrated the presence of i.v. labeled leukocytes (**Figure 4E**). However, we noted that labeled leukocytes in animal #1 (infused 5 mins before necropsy) were found in the lumen of a vessel that resembles histologically a high endothelial vein, which have recently been linked to T cell trafficking in the human decidua (62). This suggests that the 5 min infusion is capturing leukocytes that are in the decidual vasculature and possibly entering the decidua stroma (**Figure 4E**, *top left*). On the other hand, we see that labeled leukocytes in animal #2 (infused 2 hours before necropsy) seem to be embedded in the decidua stroma, suggesting that these leukocytes arrived in the decidua, crossed the endothelium, and migrated into the stroma (**Figure 4E**, *bottom left*).

## 4 Discussion

The decidua maintains a specific immunome, which dramatically remodels across pregnancy (9, 12, 63). It has been reported, in both humans and mice, that in early pregnancy there is an abundance of dNKs, while the proportion of T cells increases in late gestation (6, 55, 56). However, limited data are available on the origin of the uterine/decidual leukocytes as well as timing of leukocyte movement through the decidua, which remain controversial. Intravascular staining is a promising tool previously used to assess the traffic of leukocytes into various tissues outside of pregnancy, in both mice (47, 48, 64) and non-human primates (45, 46). Here, we demonstrate the use of intravascular staining applied to gestation-specific trafficking of these cells in and out of murine and non-human primate decidua. First, we demonstrate the feasibility of intravascular staining in the context of murine pregnancy. Our results confirmed previously reported observations of trafficking in the murine spleen (open nature of splenic blood circulation, with majority of lymphocytes labeled) and Peyer’s patches (more restricted labeling, but open to inward and outward traffic of innate and adaptive lymphocytes) (65, 66). Next, we confirmed that the injected sub-saturating antibodies act as a pulse/chase design, with no or minimal free antibody available to bind additional leukocytes *ex vivo*. This also demonstrates the stability of staining of cells *in vivo*, with no detectable dissociation of antibody from labeled cells.

In agreement with prior studies, we find a clear contribution of circulating lymphocytes to the decidual immune compartment during pregnancy. We note a dramatic increase in the proportion of IVas+ (CC + RE) leukocytes, which, considered anatomically, implies an increased proportion of immune cells in the decidual vasculature or loosely attached to the inside of the decidual vasculature in mid to late pregnancy. While we cannot know if those cells are in the process of influx or efflux from decidual stroma based on this label alone, we note the presence of increased RI leukocytes in mid-late gestation, which argues that at least part of the CC+RE cells seen are “caught” in the act of entering the decidual compartment. This influx may contribute to decidual immune reconstruction near parturition (9, 26). Furthermore, our results are in line with the observation (24) that pregnancy orchestrates the waves of migration and residency of ILCs (including NK) subsets at different stages, and that at least a subset of lymphocytes are tissue resident.

We previously demonstrated a high level of heterogeneity among decidual immune cells driving a decidual immune signature in mouse (9) and human (7) systems. In this study, we used intravascular staining to track T cell and ILC dynamics across murine gestation. We noted the proportional decrease in IVas- (RI + TL) T cells during gestation, while CC T cells increased near parturition. These findings support the observation that CD3+ T cells and mediators are essential in creating a specific microenvironment that plays an important role in spontaneous labor at term pregnancy (55, 67, 68). Decidual NK cells (dNKs), as part of the broader ILC compartment, are the major and temporally regulated decidual inhabitants relevant to understanding pregnancy pathology. Our previous analysis of murine decidual cells demonstrated considerable heterogeneity amongst dILC subsets across pregnancy—from first trimester through term decidua (26). Like T cells, TL dILCs showed a dramatic reduction towards mid pregnancy. Interestingly, the majority of TL ILCs are NK1.1-CD49b- (ILC1-like), with this same population representing a greater proportion of RI ILCs as gestation continues (**Figure 3C**), suggesting that these cells are present at pregnancy initiation and are recruited as pregnancy continues. Furthermore, our clustering analysis highlighted the presence CC ILCs (Cluster 3), which increased in proportion across pregnancy, suggesting that these ILCs might be recruited near parturition in a similar fashion as T cells.

The integrins CD49a and CD103 have been identified as markers essential for resident memory T cells (T_RM_) maintenance and motility (69) and are expressed by tissue-resident NKs and ILC1s (52, 70). In T_RM_, both CD49a and CD103 are necessary for tissue retention (71, 72) and can help shape effector functions (73). Coincidentally, CD49a expression by dNKs has been linked to lower cytotoxic functions (74). TGFβ is an important factor in upregulating expression of CD49a and CD103 in both ILCs and T cells (75–77). In pregnancy, TGFβ is an important immune modulator in the decidua (78) and *ex vivo* treatment of peripheral NK cells with decidual stroma-derived TGFβ promotes transition to a dNK phenotype (79). This, together with our findings suggest that ILCs are recruited to the decidua and gain residency markers (CD49a and CD103) upon entrance under the influence of the unique decidual cytokine milieu.

The murine pregnancy model has the advantages of a short gestational length and significant literature and is a great tool to answer mechanistic questions using genetic manipulations. However, there are important physiological differences between murine and human pregnancy, including placental morphology and degree of placental invasiveness. The macaque represents a more faithful model of human pregnancy, sharing similar characteristics, including extensive spiral artery remodeling (80). To determine the potential of the macaque to understand how leukocytes traffic through the maternal-fetal interface, we leveraged intravascular staining, a technique recently applied in the macaque (45). Although we employed single infusions, this feasibility study shows the potential of identifying leukocytes that have recently entered the decidua as opposed to those that are resident in the stroma. This was confirmed by the spatial localization of labeled leukocytes in macaque decidua indicating temporal progression in migration of leukocytes from the decidual vasculature to deeper stroma. These findings highlight the importance of coupling flow cytometry results with imaging, when possible, as these will better inform our understanding of leukocyte trafficking in the decidua across primate pregnancy. Of course, any interpretation from serial intravascular staining experiments, both in the mouse and NHP models, should be limited to the window in which antibody infusions are done and any conclusions beyond that timepoint would need further confirmation. That is to say, assigning tissue residency to leukocytes identified as tissue localized would require more evidence (such as expression of residency markers) than that obtained simply by the lack of i.v staining.

As with most approaches, intravascular labeling has challenges. In mice, a major hurdle was the frequency of pseudopregnancies. At early gestation (GD6-8), we relied on the presence of a vaginal plug to determine whether mating had occurred. However, because pregnancy could not be confirmed this early in gestation, we had many animals assigned to the early gestation timepoint that were pseudo-pregnant. This led to the loss of animals and reagents (i.e infused antibodies) which restricted our ability to gather more replicates. In the NHP model, the major hurdle is expense. Macaques are larger thus require higher amounts of antibody to reach adequate saturation levels. Lastly, flow cytometry panel design and degree of immunophenotyping could be restricted as at least three channels need to be devoted to CD45 detection.

Overall, our data show how serial intravascular staining is a valuable tool in expanding our understanding of decidual leukocyte traffic during pregnancy. We propose that this technique will prove useful in answering outstanding questions regarding leukocyte traffic in the decidua, including transcriptional regulation dictating entrance/exit into and out of the decidua. Moving forward, similar studies can include a longer time frame between infusions and/or additional infusions to obtain better temporal resolution within waves of decidual leukocyte traffic. Future studies can also include assessment of local proliferation rates of decidual leukocytes and both chemokines and adhesion molecules involved thus providing a complete picture of leukocyte population dynamics at the maternal-fetal interface.

## Data availability statement

The raw data supporting the conclusions of this article will be made available by the authors, without undue reservation.

## Ethics statement

The animal study was approved by University of Wisconsin Institutional Animal Care and Use Committee. The study was conducted in accordance with the local legislation and institutional requirements.

## Author contributions

JV: Conceptualization, Formal Analysis, Investigation, Methodology, Writing – original draft. MM: Writing – original draft. SB: Investigation, Writing – review & editing. LK: Investigation, Writing – review & editing. MK: Investigation, Writing – review & editing. FJ: Investigation, Writing – review & editing. RF: Investigation, Writing – review & editing. EB: Investigation, Writing – review & editing. TG: Conceptualization, Funding acquisition, Methodology, Resources, Supervision, Writing – review & editing. AS: Conceptualization, Funding acquisition, Methodology, Resources, Supervision, Writing – review & editing.
