# Corrigendum: Deciphering decidual leukocyte traffic with serial intravascular staining

**DOI:** 10.3389/fimmu.2024.1378417

**Published:** 2024-02-26

**Authors:** Jessica Vazquez, Mona A. Mohamed, Soma Banerjee, Logan T. Keding, Michelle R. Koenig, Fernanda Leyva-Jaimes, Rachel C. Fisher, Emily M. Bove, Thaddeus G. Golos, Aleksandar K. Stanic

**Affiliations:** ^1^ Department of Obstetrics and Gynecology, University of Wisconsin-Madison, Madison, WI, United States; ^2^ Wisconsin National Primate Research Center, Madison, WI, United States; ^3^ Department of Comparative Biosciences, University of Wisconsin-Madison, Madison, WI, United States

**Keywords:** decidua, leukocytes, traffic, gestation, non-human primate, murine, T cells, ILCs

In the published article, there was an error in the Funding statement. Authors mistakenly omitted funding sources and read as follows: “This research was supported by NIH grants T32 HD1013840 to J.V., T32 GM007133 to M.R.K., R21AI175753 to T.G.G and A.S., and P51 OD011106 to the Wisconsin National Primate Research Center. The content is solely the responsibility of the authors and does not necessarily represent the official views of the NIH.”

The correct Funding statement appears below.

