# Supplementary material for: Deciphering decidual leukocyte traffic with serial intravascular staining
Source: Front Immunol. 2024 Jan 10;14:1332943. doi: 10.3389/fimmu.2023.1332943 (PMC10806228; doi:10.3389/fimmu.2023.1332943)
Supplement: Supplementary file 1 [file DataSheet_1.docx]

Supplementary Material

Deciphering Decidual Leukocyte Traffic with Serial Intravascular Staining

Jessica Vazquez^1,2^, Mona A. Mohamed^1^, Soma Banerjee^1^, Logan Keding^1,2^, Michelle Koenig^3^, Fernanda Leyva-Jaimes^1,2^, Rachel C. Fisher^1^, Emily Bove^2^, Ted Golos^1,2,3^, Aleksandar K. Stanic^1^*****

^1^Department of Obstetrics and Gynecology, University of Wisconsin-Madison, Madison, WI, USA

^2^Wisconsin National Primate Research Center, ^3^Department of Comparative Biosciences, University of Wisconsin-Madison, Madison, WI, USA

*** Correspondence:**A.K. Stanic

Department of Obstetrics and Gynecology

University of Wisconsin-Madison

Perinatal Research, Meriter-park

202 S. Park Street, Madison, WI 53715, USA

E-mail: [stanickostic@wisc.edu](mailto:stanickostic@wisc.edu)

**Supplementary Figure 1 Representative Gating Scheme Identifying T cells and ILCs.** *Top* Identification of T cells and ILCs in murine decidua. *Bottom* Determination of circulatory status of T cells and ILCs.

**Supplementary Figure 2 Quantification of RI and TL lymphocytes across gestation.** Proportion of RI and TL T cells (top) and ILCs (bottom) in murine decidua within IVas-.

**Supplementary Figure 3 FlowSOM clustering and tSNE visualization.** ILCs (pre-gated LiveCD45+CD3-TCRβ-CD11c-CD19-Ly6G-) from 2 virgin, 2 GD6-8 (Early), and 2 GD12-18 (Mid/Late) animals were clustered using FlowSOM (top) and visualized with t-SNE (bottom). *Top*, FlowSOM tree showing the 8 metaclusters. *Bottom,* t-SNE visualization partitioned by FlowSOM clusters.

**Supplementary Table 1 Antibodies used for flow cytometry analysis.**

| **Marker** | **Fluorochrome** | **Clone** | **Cytometer** | **Target Species** |
| --- | --- | --- | --- | --- |
| CD45 i.v 5 mins | AF488 | 30-F11 | Fortessa/Aurora | Mouse |
| CD45 i.v 24 hours | AF647 | 30-F11 | Fortessa/Aurora | Mouse |
| CD45 | PE-Cy5 | 30-F11 | Fortessa | Mouse |
| CD103 | BV711 | 2E7 | Aurora | Mouse |
| CD11c | AF700 | HL3 | Fortessa/Aurora | Mouse |
| CD19 | AF700 | 1D3 | Fortessa/Aurora | Mouse |
| CD3e | AF700 | 500A2 | Fortessa | Mouse |
| CD3e | Pacific Blue | 500A2 | Aurora | Mouse |
| CD4 | Pe-Fire700 | GK1.5 | Aurora | Mouse |
| CD49a | BUV737 | Ha31/8 | Aurora | Mouse |
| CD49b (DX5) | BV650 | HMa2 | Fortessa | Mouse |
| CD49b (DX5) | BUV661 | HMa2 | Aurora | Mouse |
| Eomes | eFluor450 | Dan11mag | Fortessa | Mouse |
| KLRG1 | APC-eFluor780 | 2F1 | Aurora | Mouse |
| Ly6G | AF700 | RB6-8C5 | Fortessa/Aurora | Mouse |
| NK1.1 | PE-Cy7 | PK136 | Fortessa | Mouse |
| NK1.1 | BUV395 | PK136 | Aurora | Mouse |
| TCRB | AF700 | H57-597 | Fortessa | Mouse |
| TCRb | Pacific Blue | H57-597 | Aurora | Mouse |
| CD45 i.v | AF555 | D058-1283 | Aurora | NHP |
| CD45 | BUV395 | D058-1283 | Aurora | NHP |

**Supplementary Table 2 Classification of clusters identified by FlowSOM.**

| **Cluster** | **Phenotype** | **Classification** |
| --- | --- | --- |
| 1 | KLRG1+NK1.1-CD49b-CD49a+CD103-CD4- | ILC2-like |
| 2 | KLRG1dimNK1.1-CD49b-CD49adimCD103-CD4- | ILC2-like |
| 3 | KLRG1-NK1.1-CD49b-CD49a-CD103-CD4- | ILCs |
| 4 | KLRG1-NK1.1dimCD49bdimCD49adimCD103-CD4- | intILC1 |
| 5 | KLRG1-NK1.1+CD49b+CD49a-CD103-CD4- | NK |
| 6 | KLRG1-NK1.1-CD49b-CD49a-CD103-CD4+ | ILC3-like |
| 7 | KLRG1-NK1.1-CD49b-CD49a-CD103+CD4- | ILC1 |
| 8 | KLRG1-NK1.1-CD49b-CD49a-CD103+CD4- | ILC1 |
